# A Guide to Transient Expression of Membrane Proteins in HEK-293 Cells for Functional Characterization

**DOI:** 10.3389/fphys.2016.00300

**Published:** 2016-07-19

**Authors:** Amanda Ooi, Aloysius Wong, Luke Esau, Fouad Lemtiri-Chlieh, Chris Gehring

**Affiliations:** ^1^Division of Biological and Environmental Sciences and Engineering, King Abdullah University of Science and TechnologyThuwal, Saudi Arabia; ^2^Institute of Integrative Biology of the Cell, Centre National de la Recherche Scientifique, Le Commissariat à l'Energie Atomique et aux Energies Alternatives, Paris-Sud UniversityGif-Sur-Yvette, France

**Keywords:** transfection, heterologous expression, membrane proteins, human embryonic kidney 293 cells, fluorescent imaging, electrophysiology

## Abstract

The human embryonic kidney 293 (HEK-293) cells are commonly used as host for the heterologous expression of membrane proteins not least because they have a high transfection efficiency and faithfully translate and process proteins. In addition, their cell size, morphology and division rate, and low expression of native channels are traits that are particularly attractive for current-voltage measurements. Nevertheless, the heterologous expression of complex membrane proteins such as receptors and ion channels for biological characterization and in particular for single-cell applications such as electrophysiology remains a challenge. Expression of functional proteins depends largely on careful step-by-step optimization that includes the design of expression vectors with suitable identification tags, as well as the selection of transfection methods and detection parameters appropriate for the application. Here, we use the heterologous expression of a plant potassium channel, the *Arabidopsis thaliana* guard cell outward-rectifying K^+^ channel, AtGORK (At5G37500) in HEK-293 cells as an example, to evaluate commonly used transfection reagents and fluorescent detection methods, and provide a detailed methodology for optimized transient transfection and expression of membrane proteins for *in vivo* studies in general and for single-cell applications in particular. This optimized protocol will facilitate the physiological and cellular characterization of complex membrane proteins.

## Introduction

Mammalian cells such as the human embryonic kidney 293 (HEK-293) and the Chinese hamster ovary (CHO) cells are widely used as hosts to express recombinant proteins to study their structural, biophysical, and pharmacological properties (Baldi et al., 2007; Dalton and Barton, 2014). HEK-293 cells in particular are an attractive heterologous system for expression of membrane proteins not least because they have post-translational modification machineries required for the proper folding and/or optimal biological activity of target proteins. They also exhibit high transfection efficiency, faithful translation, and processing of proteins (Wurm, 2004) that will result in higher protein yields (Backliwal et al., 2008a) as compared to other mammalian cells, e.g., CHO cells (Bollin et al., 2011). These attributes together with the cell size, morphology, rapid division rate, the ease of maintenance, and the low expression of native channels as well as the capacity to express transgenic receptor proteins and ion channels with high fidelity (Thomas and Smart, 2005), have established HEK-293 cells as a host of choice for transient heterologous expression of membrane proteins for structural studies (Nettleship et al., 2008; Chaudhary et al., 2011; Andrell and Tate, 2013), biopharmaceutical (Thomas and Smart, 2005; Jager et al., 2013), and electrophysiology applications (Lemtiri-Chlieh and Ali, 2013).

Despite these advantages, high-level expression of complex membrane proteins such as ion channels and trans-membrane receptors originating from a different species for current-voltage measurements has remained a challenge (Gan et al., 2006; Allen et al., 2009). For electrophysiology in particular, a high expression of proteins is critical for single-cell current recordings in the whole-cell mode. Membrane protein biosynthesis in the host is limited by the different composition of lipid bilayers between human and other species that may prevent proper folding of expressed proteins into their functional native three-dimensional conformations. As such, optimization of plasmids, culture media, growth conditions, or combinations thereof have been undertaken in the past to enhance the expression of membrane proteins (for review, see Jäger et al., 2015). These include the introduction of mild hypothermia (Wulhfard et al., 2008; Lin et al., 2015) and the addition of inhibitors of histone deacetylase to the culture media (Fan et al., 2005; Backliwal et al., 2008b). In addition to the essential elements required for the expression of recombinant protein, vectors can also be synthetically engineered to include optimized introns and codon usage (Gustafsson et al., 2004) and post-transcriptional regulatory elements (Mariati et al., 2010) to increase yields by stabilizing recombinant transcripts.

The delivery of the recombinant vector into the host cell and the detection of expressed proteins are the two critical stages crucial to the expression and study of recombinant proteins in HEK-293 cells. Recombinant proteins expressed in transiently transfected cells can be identified with a method that requires the simultaneous co-transfection of a lymphocyte surface marker antigen (CD8-alpha) or a fluorescent protein plasmid in addition to a second expression vector that contains the gene of interest (Lemtiri-Chlieh and Ali, 2013). This method enables visual identification of individual cells decorated with anti-CD8 antibody coated polystyrene beads (Jurman et al., 1994; Fortin and Hugo, 1999) under the light microscope or fluorescence detection of cells co-expressing the fluorescent proteins (Bestvater et al., 2002; Lin et al., 2015) and has been used for the study of ion channels and receptor proteins including the mammalian N-methyl-D-aspartate (NMDA; Ehlers et al., 1996) and α-amino-3-hydroxy-5-methyl-4-isoxazolepropionic acid (AMPA) receptors (Swanson et al., 1997; Lin et al., 2015), the *Arabidopsis thaliana* cyclic nucleotide-gated ion channels (AtCNGCs) (Leng et al., 2002; Hua et al., 2003) and Arabidopsis K^+^ transporters (AKTs) (Lacombe et al., 2000; Cherel et al., 2002). While simple, rapid and inexpensive, expression of the CD8-alpha marker antigen or the fluorescent protein in the transfected cells has no direct correlation with the transfection efficiency and expression of the recombinant protein as the success rate of the introduction of both these marker and target gene expression vectors into the cells may be highly variable. Recent methods using fluorescent tags such as a dye-sensitive epitope (Tour et al., 2003; Rudner et al., 2005) or a fluorescent protein fusion (Snapp, 2005) provide a direct and better correlation between fluorescent signal and transfection efficiency and protein expression level although fusion tags such as fluorescent proteins may cause structural constrains that interfere with protein function. These fluorescent detection approaches have enabled successful expression of a number of membrane proteins including the connexin-43 (Gaietta et al., 2002), the AMPA receptors (Ju et al., 2004), the G protein-coupled receptors (Hoffmann et al., 2005) and the human ether-a-go-go-related gene (hERG) channel (Claassen et al., 2008; Huang et al., 2011) that were subsequently used for protein localization and trafficking as well as current-voltage measurement studies.

Although the expression of several membrane proteins and ion channels in HEK-293 cells have been reported previously, a detailed authoritative protocol that describes the key stages in the transient transfection and in-cell detection of recombinant proteins optimized for single-cell applications, is currently lacking. Here, we use the transient expression of an *A. thaliana* guard cell outward-rectifying K^+^ channel, AtGORK (At5G37500) in HEK-293 cells as an example to assess current commonly used transfection reagents and the fluorescent detection methods, and provide a specific protocol that is easily accessible for the general expression of membrane proteins in HEK-293 cells suitable for biological characterization. As an example, AtGORK represents: (1) a difficult to express multi-pass membrane protein, (2) originates from a different species, and (3) needs to assemble into a heteromeric complex to achieve functionality. These three characteristics can hamper optimal expression of membrane proteins in HEK cells. In addition to this authoritative step-by-step protocol, we also include cautionary measures, and propose optimization strategies and recommendations extendable and amendable for different applications or proteins.

## Materials and equipment

### Cell line

- Human Embryonic Kidney 293 (293FT) cell line (Cat. no. R70007, Life Technologies, Carlsbad, CA). The 293F cell line is a fast-growing variant of the 293 cell line originally obtained from Robert Horlick at Pharmacopeia while the 293T cell line is a variant of 293 cells that harbors the SV40 large T antigen which can bind to SV40 enhancers of expression vectors to increase protein production. Here, we use the 293FT cell line to leverage on both the “fast growing” and “increased protein production” benefits.

### Culture media

- Dulbecco's Modified Eagle's medium, DMEM (1X) + GlutaMAX™-I (Cat. no. 31966021, Life Technologies Europe BVM, Bleiswijk, Netherlands).
- 10% (v/v) Fetal Bovine Serum (Cat. no. 16000044, Life Technologies, Carlsbad, CA).
- 1 (v/v) % Penicillin-Streptomycin (10,000 U/mL; Cat. no. 15140122, Life Technologies Europe BVM, Bleiswijk, Netherlands).
- Opti-MEM^®^ I Reduced Serum medium (Cat. no. 31985062, Life Technologies, Carlsbad, CA).
- Freezing medium consisting of 95% (v/v) complete medium and 5% (v/v) DMSO.

### Cloning cells

- One Shot^®^ Mach1™ T1 phage-resistant chemically competent *E. coli* cells (Cat. no. C862003, Life Technologies, Carlsbad, CA).

### Vectors

- Gateway^®^ pDONR™221 Vector (Cat. no. 12536017, Life Technologies, Carlsbad, CA).
- pcDNA™6.2/cLumio™-DEST (Cat. no. 12589016, Invitrogen Corporation, Carlsbad, CA).
- pcDNA™6.2/nLumio™-*GW/p64* (Cat. no. 12589016, Invitrogen Corporation, Carlsbad, CA).
- Vivid Colors™ pcDNA™6.2/EmGFP-DEST Gateway^®^ vector (Cat. no. V35520, Life Technologies, Carlsbad, CA).
- Vivid Colors pcDNA6.2/C-terminal tagged *EmGFP/GW/CAT* plasmids (Cat. no. V35520, Life Technologies, Carlsbad, CA).

### Chemicals/reagents

- Lipofectamine^®^ 2000 (Cat. no. 11668019, Life Technologies, Carlsbad, CA).
- Lipofectamine^®^ 3000 (Cat. no. L3000015, Life Technologies, Carlsbad, CA).
- FuGENE^®^ HD formulation (Cat. no. E2311, Promega, Madison, WI).
- 0.25% Trypsin-EDTA solution (Cat. no. T4049, Sigma-Aldrich Chemie GmbH, Steinheim, Germany).
- 0.4% Trypan Blue stain (Cat. no. T10282, Life Technologies, Carlsbad, CA).
- 50 μg/mL poly-D-lysine hydrobromide (Cat. no. P0899, Sigma-Aldrich, St. Louis, MO).
- Gateway^®^ LR Clonase^®^ II Enzyme Mix (Cat. no. 11791100, Invitrogen, Carlsbad, CA).
- Invitrogen™ PureLink^®^ HQ Mini Plasmid Purification kit (Invitrogen™, ThermoFisher Scientific, Carlsbad, CA).
- LB broth (Lennox) (Cat. no. L3022, Sigma-Aldrich Chemie GmbH, Steinheim, Germany).
- Carbenicillin disodium salt (Cat. no. C3416, Sigma-Aldrich Chemie GmbH, Steinheim, Germany).
- Lumio™ Green In-Cell Detection kit (Cat. No. 12589057, Life Technologies, Carlsbad, CA).
- 10X Phosphate Buffered Saline (PBS) solution (Cat. No. P5493, Sigma-Aldrich, St. Louis, MO).
- TRIzol^®^ reagent (Cat. No. 15596026, Life Technologies, Carlsbad, CA).
- Applied Biosystems™ High-Capacity cDNA Reverse Transcription Kit (Cat.no. 4368814, Life Technologies, Carlsbad, CA).
- KAPA Taq PCR kit (Cat. no. KR0352, KAPA Biosystems, Wilmington, MA).
- Dimethyl sulfoxide (Cat. no. D8418, Sigma-Aldrich Chemie GmbH, Steinheim, Germany).
- Acetone (Cat. no. 320110, Sigma-Aldrich, St. Louis, MO).
- Absolute ethanol (Cat. no. 32205, Sigma-Aldrich, St. Louis, MO).
- Chloroform (Cat. no. 528730, Sigma-Aldrich Chemie GmbH, Steinheim, Germany).
- 2-Propanol (Cat. no. I9516, Sigma-Aldrich Chemie GmbH, Steinheim, Germany).
- DEPC-treated water (Cat. no. 95284, Sigma-Aldrich Chemie GmbH, Steinheim, Germany).

### Buffer preparation

- Intracellular solution consisting of 100 mM KCl, 25 mM N-methyl-D-glucamine, 10 mM HEPES, 10 mM EGTA, 1 mM CaCl_2_, and 4 mM MgCl_2_ at pH 7.30 and at 280 ± 5 mOsm (osmolarity is adjusted by adding sorbitol). This solution is prepared a day before the patch-clamp experiment, filter sterilized through a 0.22 μm Stericup-GP and stored at 4°C.
- External bath solution contained 10 mM KCl, 145 mM N-methyl-D-glucamine, 20 mM HEPES, 10 mM glucose, 0.5 mM CaCl_2_, and 1 mM MgCl_2_ at pH 7.30 and at 310 ± 5 mOsm (osmolarity is adjusted by adding sorbitol). This solution is prepared a day before the patch-clamp experiment, filter sterilized through a 0.22 μm Stericup-GP and stored at 4°C.

### Apparatus/equipment

- 75 cm^2^, 250 mL Cellstar^®^ cell culture flask (T-75; Cat. no. 658175, Greiner Bio-One GmbH, Frickenhausen, Germany).
- Corning^®^ Costar^®^ 6 well cell culture plates (Cat. no. 64705-01, Corning Inc., Brooklyn, NY).
- 15 mL Falcon™ conical centrifuge tubes (Cat. no. 14-959-70C, Corning Inc., Brooklyn, NY).
- Cryovials (Thermo Fisher Scientific, Waltham, MA).
- Stericup-GP, 0.22 μm, polyethersulfone, radio-sterilized (Cat. no. SCGPU05RE, Merck Millipore, Danver, MA).
- Countess^®^ II Automated Cell Counter (Cat. no. AMQAX1000, Life Technologies, Carlsbad, CA).
- 5004 MICRO-OSMETTE™ automatic high sensitivity 50 μL osmometer (PSi Precision Systems Inc., Natick, MA).
- Fisherbrand™ 12 mm circle cover glasses (Cat. no. 12-545-80, Fisher Scientific, Marietta, OH).
- Thick/standard wall borosilicate glass capillaries (Cat. no. B150-86-10, Sutter Instrument^®^, Novato, CA).
- Fluorescence microscope (Nikon Eclipse TS100, Melville, NY).
- P-1000 FLAMING/BROWN micropipette puller (Sutter instrument, Novato, CA).
- Microforge (Cat. no. MF-830, Narishige Group, Japan).
- Inverted microscope Carl Zeiss Axio Observer.A1 (Carl Zeiss, Oberkochen, Germany).
- MultiClamp™ 700B microelectrode amplifier (Axon Instruments, Molecular Devices, Sunnyvale, CA).
- Veriti^®^ 96-Well Thermal Cycler (Cat. no. 4375786, Applied Biosystems™, ThermoFisher Scientific, Carlsbad, CA).
- New Brunswick™ Excella^®^ E23/E24R benchtop incubator shaker (Eppendorf AG, Hamburg, Germany).
- 5% carbon dioxide (CO_2_) humidified growth incubator (Series CB, BINDER, Tuttlingen, Germany).
- Water bath (VWR International, Radnor, PA).
- Precision™ compact ovens (Thermo Fisher Scientific, Marietta, OH).
- Nanodrop (Thermo Fisher Scientific, Marietta, OH.

## Procedure

### Design of plasmid construct (2–3 days)

1. Both pcDNA™6.2/cLumio™-DEST and Vivid Colors™ pcDNA™6.2/EmGFP-DEST Gateway^®^ vector (Figure S1) are selected as the expression vectors to heterologously express the full-length *A. thaliana* GORK channel (AtGORK) (At5G37500) in HEK-293 cells.
2. The Lumio™ tag consisting of 6 amino acids is located at 27 amino acid residues downstream of the C-terminal of AtGORK (Figure S1A). The Lumio™ tag contains a tetra-cysteine motif (Cys-Cys-Pro-Gly-Cys-Cys) that forms an arsenical hairpin detectable by interactions with biarsenical labeling reagents (e.g., Lumio™ Green or Lumio™ Red; Griffin et al., 1998; Adams et al., 2002).
3. The Emerald Green Fluorescent Protein (EmGFP) derived from *Aequorea victoria* GFP (Tsien, 1998) is C-terminally tagged at the end of the AtGORK sequence in the mammalian expression clone (Figure S1B). ❖ Troubleshooting (**Table 2**).
4. Perform codon optimization of *AtGORK* sequences using the GeneOptimizer^®^ software. ♦ CRITICAL STEP This step maximizes the expression of AtGORK in the mammalian expression system. ❖ Troubleshooting (**Table 2**).
5. Assemble the codon optimized *AtGORK* sequences from synthetic oligonucleotides and/or PCR products using the GeneArt™ Gene Synthesis service offered by ThermoFisher Scientific. • OPTIONAL Alternatively, this step can be carried out using the conventional PCR cloning method.

### Cloning, transformation and plasmid purification (4–5 days)

6. Clone the optimized *AtGORK* fragment into a Gateway^®^ pDONR™221 entry vector.
7. Perform a LR recombination reaction between the entry vector and the pcDNA™6.2/cLumio™-DEST or the Vivid Colors™ pcDNA™6.2/EmGFP-DEST Gateway^®^ vector using the Gateway^®^ LR Clonase^®^ II Enzyme Mix following the manufacturer's protocol.
8. Transform the pcDNA™6.2/cLumio™-DEST and Vivid Colors™ pcDNA™6.2/EmGFP-DEST Gateway^®^ expression vectors containing the *AtGORK* insert into One Shot^®^ Mach1™-T1^R^ phage-resistant chemically competent *E. coli* cells following the manufacturer's transformation procedure.
9. Inoculate a single, overnight colony in 5 mL of selective LB broth containing 50 μg/mL carbenicillin and shake at 37°C overnight before isolating the plasmid.
10. Isolate and purify high quality plasmid DNA using the Invitrogen™ PureLink^®^ HQ Mini Plasmid Purification kit according to the manufacturer's instructions. ♦ CRITICAL STEP This step provides a good yield of high-quality plasmid DNA for use in mammalian cell transfections.
11. Analyze both the plasmid constructs by sequencing to verify that the insertion of *AtGORK* into the expression vectors is in-frame and error-free.

### Growth and maintenance of the 293FT cell line (2–3 days)

♦ CRITICAL STEP Culture media and trypsin-EDTA solution should be pre-warmed in the water bath at 37°C.

■ CAUTION Handle the cell line as potentially bio-hazardous material under at least Biosafety Level 2 containment.

12. Remove the vial of frozen cells from −140°C freezer and thaw quickly in a 37°C water bath.
13. Transfer the cells to T-75 cm^2^ culture flask containing 11 mL of complete medium consisting of Dulbecco's modified Eagle's medium, DMEM (1X) + GlutaMAX™-I supplemented with 10% (v/v) fetal bovine serum and 1% (v/v) penicillin-streptomycin (10,000 U/mL). ❖ CRITICAL STEP Ensure that all solutions and equipment that come in contact with the cells are sterile by performing the work in a laminar flow hood and always practice good aseptic technique.
14. Maintain the cell culture at 37°C in a 5% CO_2_ humidified growth incubator. Replace with fresh, complete medium 24 h after seeding. ♦ CRITICAL STEP Replacement with fresh, complete medium upon overnight seeding is important to remove residual DMSO contained in the freezing medium. ■ CAUTION DMSO is toxic to cell growth.
15. Incubate the cells until they are 80–90% confluent. ♦ CRITICAL STEP Pass the 293FT cells when they are ≥80% confluent to avoid cell overgrowth.

### Transient transfection and heterologous expression of AtGORK in 293FT cells (1 day)

♦ CRITICAL STEP We recommend using early-passage cells for transfection experiments. Cells should be at the appropriate confluence and at >90% viability prior to transfection.

16. Remove all medium from the flask and add 2 mL of 0.25% trypsin-EDTA solution to the adherent monolayer and incubate 1–5 min at 37°C in a 5% CO_2_ humidified growth incubator until the cells detach. Check the cells under a microscope to confirm that most of the cells have been detached from the bottom surface of the flask. Increase the incubation time if the cells are still attached.
17. Add 6 mL of complete medium to the cell suspension and mix gently. ♦ CRITICAL STEP This step is to inactivate the trypsin activity as serum contains inhibitors of trypsin.
18. Determine cell viability using trypan blue exclusion and total cell count using a cell counter (e.g., Countess^®^ II Automated Cell Counter).
19. Dilute the cell density to a total of 2.5 × 10^5^ viable cells/2 mL in pre-warmed complete medium for the transfection protocol. • OPTIONAL the final cell density for transfection protocol varies depending on the purpose of experiment. Here, we recommend an optimal cell density of 2.5 × 10^5^ viable cells/2 mL in order to select for single cell expressing the channel protein for current-voltage measurements. ❖ Troubleshooting (**Table 2**).
20. Seed the remaining cells at a total density of 1 × 10^6^ viable cells in a T-75 cm^2^ flask containing 11 mL of pre-warmed complete medium and maintain the cells as adherent monolayer cultures at 37°C in a 5% CO_2_ incubator for cell establishment.
21. Once the cells have been established, we recommend freezing some cells for storage and for future use by freezing the cells at a density of at least 3 × 10^6^ viable cells/ mL and at >90% viability in labeled cryovials containing the freezing medium. ♦ CRITICAL STEP Prepare fresh freezing medium immediately before use.
22. Clean the 12 mm circle cover glasses by placing the coverslips in a 50 mL beaker containing acetone and swirl the beaker gently to ensure that all the coverslips are immersed in acetone. Incubate overnight in a fume hood. ■ CAUTION Acetone is a highly flammable and toxic material that should only be used in a fume hood.
23. Discard the acetone solution and wash the coverslips thrice by rinsing with absolute ethanol. Repeat step 22 by replacing acetone with absolute ethanol. ■ CAUTION Ethanol is a highly flammable and toxic solvent that should only be used in a fume hood.
24. Discard the ethanol solution and wash the coverslips thrice by rinsing with sterile water. Dry the clean coverslips by incubating in an oven at 65°C overnight. Store the clean coverslips at room temperature.
25. Place four clean coverslips in a well of a 6-well, flat bottom cell culture plate and coat the coverslips with 2 mL of 50 μg/mL poly-D-lysine hydrobromide at 37°C overnight. Discard the poly-D-lysine hydrobromide solution and wash the coated coverslips thrice with sterile water prior to transfection. ♦ CRITICAL STEP Coating of coverslips with poly-D-lysine facilitates the cell attachment to the surface of coverslips by promoting the electrostatic interactions between the cell membrane and the polyamino acids on the culture surface. ❖ Troubleshooting (**Table 2**).
26. We use cationic lipid-based transfection reagents (Felgner and Ringold, 1989), Lipofectamine^®^ 2000, Lipofectamine^®^ 3000, and non-liposomal FuGENE^®^ HD formulation to transiently transfect the 293FT cells with 2.5 μg of *AtGORK* plasmid obtained in step 10 according to the respective manufacturer's instructions (Table 1). ♦ CRITICAL STEP Equilibrate the transfection reagents to room temperature before use and mix by inverting or through a brief vortex. • OPTIONAL We recommend the use of OPTI-MEM^®^ I (1X) Reduced Serum Medium as diluent in all transfection steps. Alternatively, serum-free DMEM (1X) + GlutaMAX™-I medium can be used for the transfection protocol. ❖ Troubleshooting (Table 2).
27. The “reverse” transfection method entails adding the DNA-lipid complex in a drop-wise manner directly to the cell suspension containing total viable cells of 2.5 × 10^5^/2 mL in a 15 mL centrifuge tube. Mix gently by pipetting up and down. Transfer the cell mixture to a well of 6-well culture dish containing the coated coverslips obtained in step 25. ♦ CRITICAL STEP The DNA-lipid complex should be added in a drop-wise manner directly to the cell suspension without touching the walls of the tube prior to mixing by gentle pipetting and followed by plating and culturing overnight at 37°C in a 6-well culture dish. ❖ Troubleshooting (Table 2).
28. Here, we define “standard” transfection as a method entailing the addition of the plasmid DNA and transfection reagent mixture in a drop-wise manner to the adherent 293FT cells that have been seeded at a final viable cell concentration of 2.5 × 10^5^ a day before. ♦ CRITICAL STEP The DNA-lipid complex should be added in a drop-wise manner and distributed homogenously to the adhered cells in the culture dish by gentle swirling without disturbing the adhered cells (Figure S4B).
29. The “double” transfection refers to the combination of both “standard” and “reverse” methods in the following sequence: reverse transfection and incubates overnight, followed by standard transfection on the following day.
30. Incubate the cells at 37°C overnight in a 5% CO_2_ humidified growth incubator.

**Table 1:** **Lipid-mediated transient transfection experimental parameters and conditions**.

**Component per well**	**FuGENE^®^ HD**	**Lipofectamine^®^**	
		**2000**	**3000**
293FT cell number/ 2 mL	2.5 × 105 cells	2.5 × 105 cells	2.5 × 105 cells
**(i) Transfection reagent**			
OPTI-MEM^®^ I medium	100 μL	125 μL	125 μL
Transfection reagent	10 μL	12.5 μL	7.5 μL
**(ii) DNA component**			
OPTI-MEM^®^ I medium	Up to 100 μL	Up to 125 μL	Up to 125 μL
Plasmid DNA	2.5 μg	2.5 μg	2.5 μg
^*^P3000™ reagent	N/A	N/A	5 μL
Incubation time at RT	15 min	5 min	5 min

* *Applicable only for transfections using the Lipofectamine^®^ 3000 reagent*.

**Table 2:** **Troubleshooting**.

**Step**	**Parameters**	**Recommendation**	**Comments**
3	Plasmid and label	Vivid Colors™ pcDNA™6.2/EmGFP-DEST Gateway^®^ vector	EmGFP improves expression of AtGORK and reduces background noise signal (Martin et al., 2005)
4	Codon optimization	GeneOptimizer^®^ software by ThermoFisher Scientific	Maximizes the expression of *AtGORK* in the mammalian expression system
19	Amount of cells	2.5 × 10^5^ cells/2 mL	Suitable seed amount to achieve well-spaced single cells
25	Coating of cover slip	Overnight coating with Poly-D-lysine	Poly-D-lysine is preferred over poly-L-lysine because poly-D-lysine is not digestible
26	Amount of plasmid	2.5 μg	Higher plasmid amount did not significantly improve transfection efficiency and may be toxic to cells (Lin et al., 2015; Figure S2)
26	Transfection reagent	Lipofectamine^®^ 3000	Lipofectamine^®^ 3000 outperforms Lipofectamine^®^ 2000 and FuGENE^®^ HD
27	Transfection protocol	“Reverse”	“Reverse” transfection protocol saves one additional day and is generally preferred for single-cell applications as compared to the “standard” transfection
34	Detection	GFP fluorescence	Requires fluorescence microscopy with fluorescence GFP filters

### *In vivo* fluorescence labeling, imaging, and quantification (60 min)

31. Use the Lumio™ Green In-Cell Labeling kit to detect the expression of AtGORK-cLumio™ in 293FT cells following the manufacturer's instructions. Label the cells transfected with *AtGORK*-cLumio™ expression vector obtained in step 30 with 1.25 μM of Lumio™ Green. Incubate the cells at 37°C for 30 min, protected from light. ■ CAUTION Lumio™ Green is a biarsenical labeling reagent, specifically recognizes and binds to the tetracysteine Lumio™ tag, which strongly fluoresces upon binding to the Lumio™ tag. Practice safe precautionary measures by wearing protective clothing, eyewear, and nitrile gloves when handling the Lumio™ Green reagent. ♦ CRITICAL STEP Prepare fresh Lumio™ Green solution just before use and keep the solution at room temperature in dark, protected from light.
32. Upon incubation, gently remove the Lumio™ labeling solution and wash cells once with OPTI-MEM^®^ I medium. Carefully remove and discard the medium.
33. Gently add 20 μM of Disperse Blue 3 solution that is supplied with the Lumio™ In-Cell labeling kit following the manufacturer's protocol to each well. ♦ CRITICAL STEP Do not remove the solution and proceed to fluorescence imaging to detect the labeled AtGORK-cLumio™ in the presence of Disperse Blue 3 solution to reduce the high background fluorescence (Adams et al., 2002). ■ CAUTION Disperse Blue 3 solution is a non-fluorescent, hydrophobic dye that may results in eye, and skin irritation. Wear protective clothing, eyewear and nitrile gloves when handling with the solution. • OPTIONAL Due to high background signal resulted from Lumio™ Green staining, a stringent washing procedure can be carried out by adding low concentrations of thiol-containing reagent using β-mercaptoethanol that competitively binds to the thiol-binding site thereby increasing the specific labeling of the Lumio™-tagged proteins (Langhorst et al., 2006).
34. Detect expression of AtGORK-EmGFP directly in 293FT cells transfected with *AtGORK-EmGFP* plasmid by fluorescence microscopy using the green fluorescence filter at the excitation wavelength from 460 to 500 nm. ♦ CRITICAL STEP Both pcDNA™6.2/nLumio™-*GW/p64* and Vivid Colors pcDNA6.2/C-terminal tagged *EmGFP/GW/CAT* plasmids are used as the positive control for transfection efficiency. ❖ Troubleshooting (Table 2).
35. Capture three bright-field images from three randomly chosen locations under a 20 × objective lens. Capture also three fluorescence images under the same viewing fields to calculate the percentage of transfection efficiency. On average, a fluorescence image contained several hundred green cells.
36. Using Image J (Schneider et al., 2012), process and analyse the fluorescence and bright-field images according to the methods described previously (Lin et al., 2015) in order to determine the transfection efficiency and the expression levels. Each fluorescence image contains 2560 × 1920 pixels and each is assigned a value ranging from 0 to 255 on an 8-bit digital scale using Image J. ♦ CRITICAL STEP Each green fluorescent cell detected represents successful transfection and protein expression.
37. We define transfection efficiency as the number of cells that have fluorescence detectable above background divided by the total number of cells calculated on the corresponding bright-field images and is expressed as a percentage.
38. We estimate the relative expression levels of the protein by measuring the fluorescent intensities (in pixels) of the green fluorescent cells with the assumption that the protein expression in each individual cell is linearly proportional to the amount of the reporter EmGFP or Lumio™ tag that the cell expressed (Lin et al., 2015).

### RNA extraction, cDNA synthesis and PCR (1-2 days)

39. Perform total RNA extraction followed by cDNA synthesis from the transfected cells obtained in step 30 as described previously (Sagar et al., 2014).
40. Gently wash the transfected cells twice with 2 mL of cold 1X PBS solution. Discard the PBS solution.
41. Add 600 μL of TRIzol^®^ reagent to the cells and incubate at room temperature for 5 min to allow cell lysis to occur. Gently transfer the cells from the 6-well culture dish to 1.5 mL microcentrifuge tubes.
42. Add 120 μL of chloroform to the lysed cells and gently invert the tubes for 15 s. Incubate on ice for 10 min. Centrifuge at 8000 rpm at 4°C for 15 min.
43. Separate the aqueous phase and transfer to new sterile 1.5 mL microcentrifuge tubes. Add 300 μL of 2-Propanol to the aqueous phase and incubate at −20°C overnight to precipitate the RNA.
44. The following day, centrifuge at 8000 rpm at 4°C for 30 min. Discard the supernatant.
45. Wash the pellet with 600 μL of 75% (v/v) ethanol. Centrifuge at 8000 rpm at 4°C for 20 min. Discard the supernatant. Air-dry the pellet by placing the tube with the lid opened under the laminar hood. Dissolve the pellet in 30 μL of DEPC-treated water.
46. Quantify the total RNA concentration using a Nanodrop. Store the RNA at −80°C.
47. For synthesis of cDNA, set up a reaction tube consisting of 3 μg of RNA obtained in step 46, 250 ng of Oligo-dT primer and 500 μM of dNTPs. Incubate the reaction at 65°C for 5 min followed by a brief centrifugation and terminates the reaction by incubating the tube on ice.
48. Set up a cDNA reaction using the Applied Biosystems™ High-Capacity cDNA Reverse Transcription Kit by adding 1X first strand reaction Buffer, 5 mM DTT, 200 units SuperScript III RT, and 40 units of RNAse to a final reaction volume of 20 μL and run the reaction following the manufacturer's protocol. Store the cDNA at −20°C.
49. Perform PCR using the KAPA Taq PCR kit, designed *AtGORK* PCR forward and reverse primers (Table 3) and PCR cycles according to the manufacturer's instructions to determine the heterologous expression of AtGORK in 293FT cells.

**Table 3:** **Primers and PCR conditions**.

**Primer name**	**Sequence (5′–3′)**	**Tm (°C)**	**No. of cycle**
*AtGORK long* forward	GGTTCAAACACAGAGAGGTTCAG	62	40
*AtGORK long* reverse	TAATTCACGCAAGCTTGACG	62	40
*AtGORK short* forward	GGATCAGAAGAGAGTGTGACGTT	62	40
*AtGORK short* reverse	CCCTTCAGAAGCCGCG	62	40

### Current-voltage recording and analysis (60 min)

50. Perform whole-cell voltage-clamp recordings from 293FT cells transfected with *AtGORK*-cLumio™ and *AtGORK-EmGFP* plasmids respectively, obtained in step 30 as described previously (Lemtiri-Chlieh and Ali, 2013).
51. Using a P-1000 FLAMING/BROWN micropipette puller, make patch-recording pipettes by pulling the pipette from thick/standard wall borosilicate glass capillaries. Place the micropipettes in the microforge holder and apply one or two brief heat pulses to smooth the pipette tip.
52. Carefully place one of the coverslips containing the transfected cells obtained in step 30 in the chamber. Find and choose a single healthy cell to patch under the inverted fluorescence microscope.
53. Perform the perfusion system by filling the external 60 mL syringes with external bath solution. Ensure that there is no air bubble entrapped in the system and that the perfusion is running smoothly at ~1–2 mL/minute. ♦ CRITICAL STEP Avoid flooding the chamber as the salt in the bath medium can precipitate and thus damage the microscope optics.
54. Fill the recording pipette prepared in step 51 with an intracellular solution and gently tap out any entrapped air bubbles. Apply positive air pressure in the pipette using 1 mL syringe and ensure that the electrode resistance is between 3 and 5 MΩ. ♦ CRITICAL STEP Air bubbles prevent closing of the electronic circuit resulting in a high resistance reading. The internal solution is slightly hypo-osmotic as compared to the bath medium to facilitate the high resistance seal formation.
55. Upon achieving whole-cell configuration, hold the cells at −52 mV.
56. Start recording the K^+^ channel current using the voltage-clamp protocol which consists of a series of 1000 ms long squared voltage depolarization between +120 to −80 mV in −20 mV decrements. ♦ CRITICAL STEP Cells transfected with pcDNA™6.2/nLumio™-*GW/p64* and Vivid Colors pcDNA6.2/C-terminal tagged *EmGFP/GW/CAT* plasmids respectively, are used as the negative control for whole-cell recording. To differentiate between intrinsic K^+^ and AtGORK currents, AtGORK conductance was estimated as the difference between steady-state currents (mean current between 900 and 1000 ms after depolarization) and the current measured in the range of 40–60 ms from the beginning of the voltage test.
57. Use the MultiClamp™ 700B microelectrode amplifier to investigate the voltage clamp and current clamp recordings. ♦ CRITICAL STEP All signals were low-pass filtered at 2 kHz before analog-to-digital conversion and were uncorrected for leakage current or capacitive transients.
58. Analyze the data with pClamp software (version 10, Axon Instruments Inc., California, USA) and express the data as mean ± standard error mean.

### Statistical analysis

59. Perform statistical analysis using the Student's *t*-test with Microsoft Excel 2010. Set the significance to a threshold of *P* < 0.05 and *n*-values represent number of biological replicates.

## Results and discussion

Here, we examine several currently used transfection reagents and in-cell fluorescent labeling and detection methods for transient expression of membrane proteins in HEK-293 cells. We provide a generally applicable transfection procedure for the expression of membrane proteins for current-voltage measurements using the *A. thaliana* guard cell outward-rectifying K^+^ channel, AtGORK (At5G37500) as an example and discuss the potential pitfalls as well as the general considerations that must be carefully noted throughout the experimental workflow.

### An AtGORK-cLumio™ tag fusion has lower transfection efficiency than AtGORK-EmGFP

**Figure 1:**
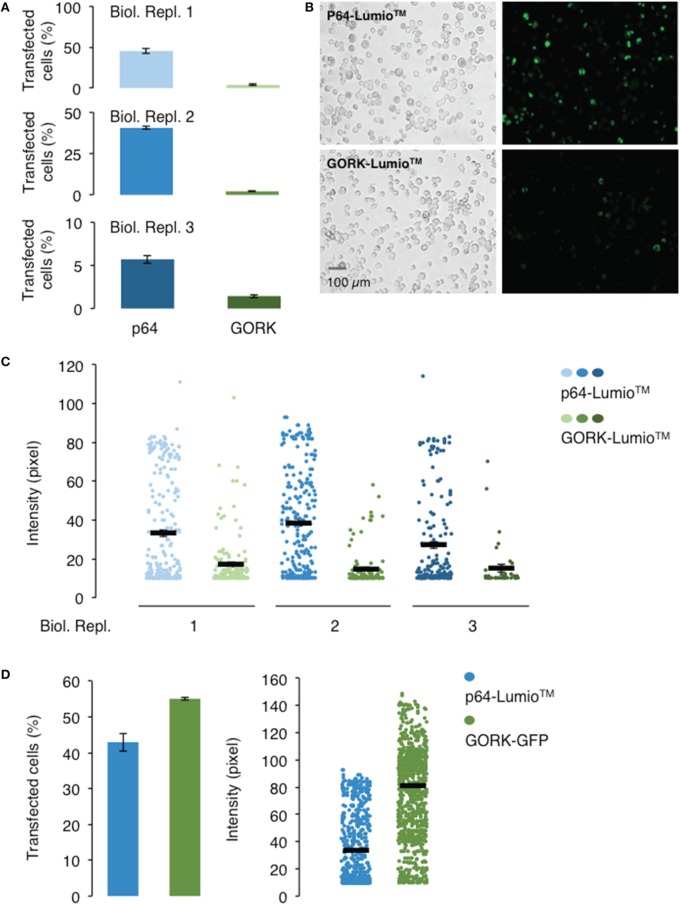
**Transfection and expression efficiencies of AtGORK tagged with Lumio™and AtGORK fused with EmGFP in HEK-293 cells. (A)** HEK-293 cells transfected with *AtGORK*-cLumio™ expression vector has reduced transfection efficiency as compared to the expression of the positive control p64 that is consistent across all three biological replicates. **(B)** Representative images showing the poor transfection efficiency of cells transfected with *AtGORK*-cLumio™ and the *p64* positive control plasmids. **(C)** The expression of AtGORK in green fluorescence cells is lower than that of p64 using the Lumio™ system as determined by their pixel intensities. **(D)** The *AtGORK-EmGFP* has a higher transfection efficiency and protein expression than that of the nLumio™-tagged *p64* in HEK-293 cells. A typical 20 × magnification of 293FT cell image contains 2560 × 1920 pixels and is converted to an 8-bit byte image based on a scale of 0–255 using ImageJ (Schneider et al., 2012). The lower limit cut-off representing appreciable and above background fluorescence, was set at 10 pixels. Transfection efficiency and protein expression of more than 100 cells in each viewing field were analyzed (see Section Procedure for analysis).

In order to select for single cell expressing the channel protein for current-voltage measurement, fluorescent labels were used as indicators for expression of the AtGORK channel in the 293FT cells. We incorporated *AtGORK* with either a Lumio™ tag (a molecular weight of 585 Da) which is a 6 amino acid long cysteine-rich tag separated by a gap of 27 amino acid residues (Figure S1A) or fused the protein with the Emerald Green Fluorescent Protein (EmGFP) of 27 kDa derived from *A. victoria* GFP at 7 amino acid residues downstream of the C-terminal of AtGORK (Figure S1B). When designing the protein linker, factors such as the length, hydrophobicity and the number of amino acid residues to yield specific structural conformations (e.g., coiled or sheet) that can mitigate structural constrains and thus protein functionality must be considered (for a comprehensive review see: (Chen et al., 2013; Chichili et al., 2013). The Lumio™ tag contains a tetracysteine motif (Cys-Cys-Pro-Gly-Cys-Cys) that forms an arsenical hairpin detectable by interactions with the membrane-permeant fluorogenic biarsenical labeling reagents (e.g., Lumio™ Green or Lumio™ Red) that strongly fluoresces upon binding, thus, enabling labeling and detection of Lumio™-tagged proteins with high specificity and affinity. Cells transfected with the manufacturer-supplied positive control plasmid pcDNA™6.2/nLumio™-*GW/p64* (Figures 1A,B) which is a nucleus-localized transcription factor (human c-myc), have poor transfection efficiency with < 50% of cells expressing the p64 protein, as indicated by the green fluorescence upon labeling with Lumio™ Green labeling reagent across all three biological replicates. However, the efficiency was reduced to < 5% for AtGORK-cLumio™ expressed in HEK-293 cells. Upon labeling with Lumio™ green reagent, the intensity of green fluorescence that correlates with the amount of p64-Lumio™ tagged protein expressed in the transfected cells is around 30–40 pixels while those cells expressing the AtGORK-cLumio™ channel has < 20 pixels (Figure 1C). Since we detected mRNA transcript of *AtGORK*-cLumio™ in the transfected cells (Figure S3), the low fluorescence intensity observed is likely due to a poor copy number of AtGORK protein expressed in the cells and/or poor detection resolution resulting from non-specific binding of the Lumio™ reagent to the cells which reduces the fluorescent signal-to-noise ratio. To reduce the high background, we performed a washing procedure using the recommended 1X Disperse Blue 3 solution. In addition, we also performed a stringent washing procedure by adding low concentrations of thiol-containing reagent using β-mercaptoethanol that competitively bind the thiol-binding site thereby increasing the specific labeling of the Lumio™-tagged proteins (Langhorst et al., 2006). These procedures however do not overcome the problem of the high background fluorescence, but reduce the specific labeling and hence the overall staining efficiency of the Lumio-tagged proteins. On the other hand, cells transfected with the *AtGORK*-Vivid Colors™ pcDNA™6.2/EmGFP-DEST Gateway^®^ construct yield a higher number of transfected cells (55%) and pixel intensity (80) (Figure 1D). This suggests firstly, that a fluorescent protein fusion enhances the expression efficiency of AtGORK channel in the HEK-293 cells and/or secondly, that the brightness of EmGFP results in better contrast in terms of resolution in detection and identification of protein of interest due at least in part to the reduced background noise signal (Martin et al., 2005). This AtGORK-EmGFP yields a two-folds higher pixel intensity than that of cells transfected with *AtGORK*-cLumio™ plasmid although the transfection efficiency is only marginally higher (1.5-fold), suggesting that in addition to an improved AtGORK protein expression in a fused GFP configuration, the detection resolution is markedly improved and this is consistent with previous reports (Martin et al., 2005) that observed a 90% improved contrast for GFP detection over biarsenic labels. This point is further supported by the fact that the expression of membrane spanning AtGORK-EmGFP channel yielded a pixel intensity that is higher than that of the soluble p64 transcription factor protein labeled with the Lumio™ system (Figure 1D). Therefore, we recommend the EmGFP-fusion configuration for the expression and identification of channel proteins in 293FT cells by fluorescence detection methods since the improved contrast is critical for single-cell selection and patch-clamp studies in addition to an improvement in the protein expression efficiency.

**Figure 2:**
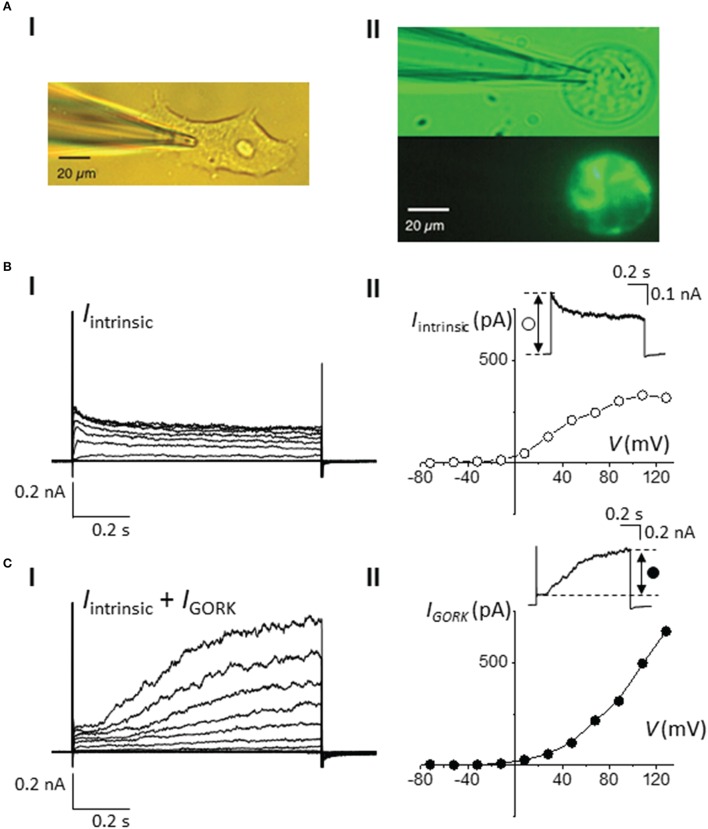
**Whole-cell current-voltage measurements of AtGORK-EmGFP in 293FT cells. (A)** Bright field and fluorescence images of (i) 293FT and (ii) *AtGORK-EmGFP*—expressing 293FT single cell at 20 × magnification field view (scale bar = 20 μm). 293FT cells were transfected with 2.5 μg of Vivid Colors™ pcDNA™6.2/EmGFP-DEST Gateway^®^ expression vector containing the *AtGORK* insert using Lipofectamine^®^ 3000 in a “reverse” format (see Section Procedure for description). **(B)** Voltage- and time-dependent (i) and I-V plot (ii) properties of the intrinsic K^+^ channels in HEK-293 cells in response to a series of depolarizing square pulse (from a holding potential, H_*V*_ = −52 mV in 20 mV increments). Inset: A current showing the fast activation and relatively slower inactivation kinetics of intrinsic K^+^ currents upon depolarization. **(C)** Whole-cell patch-clamp recordings (i) and I-V plot (ii) of intrinsic K^+^ and AtGORK channels currents, starting from H_*V*_ = −52 mV in 20 mV increments in *AtGORK-EmGFP*—expressing 293FT single cell. Inset: A representative current demonstrating the slowly activating outward currents of AtGORK upon depolarization.

Since the expression vector contains both the target gene and the fluorescent marker, cells that appear fluorescent therefore provide direct indication of the expression and localization of the target protein in the cells. This is in contrast to the co-transfection method in which the expression of the marker gene (CD8-alpha or a fluorescent protein) may not have a correlation to the success of transfection efficiency and the targeted protein expression level. However, the fluorescent tag or protein fusion may impose structural constrains that can be detrimental to the functionality of the protein of interest (Giepmans et al., 2006). Here, we generated a construct in which *AtGORK* is fused with the *EmGFP* at the C-terminal and separated by a 7 amino acid spacer. The EmGFP reporter is located at 519 amino acids away from the annotated transmembrane regions that form the channel pore at the N-terminal of AtGORK (Figure S1B), and this presumably provides a sufficient spatial separation to avoid structural constrains that may interfere with the biological activity of the channel protein. To validate the functionality of the channel activity of AtGORK, we selected single green fluorescent cells for current-voltage measurements and show that the typical transmembrane whole-cell current recordings of AtGORK (*I*_GORK_) were obtained (Figure 2). Current-voltage relationship (from +120 to −80 mV in -20 mV increments) profiles were generated from the recorded *I*_GORK_ (Figure 2Cii). We also measured currents for non-transfected, single HEK-293 cells (Figure 2B). These intrinsic currents were shown to be a mixed population of outward-rectifying K^+^ channel endogenous to the HEK-293 cell (Jiang et al., 2002). Though, they are easily recognizable, as they tend to show fast activation and relatively slower inactivation kinetics upon depolarization. On the other hand, *I*_GORK_ measured from the cells transfected with *AtGORK-EmGFP* plasmid demonstrate channel activation with distinctive kinetics (Figure 2C) which are comparable to the known delayed outwardly-rectifying K^+^-current recorded from either *A. thaliana* guard protoplasts or from *X. laevis* oocytes expressing AtGORK (Ache et al., 2000; Hosy et al., 2003). One can therefore easily distinguish the true voltage and time-dependent activated *I*_GORK_ from the other native currents (Figures 2B,C). Therefore, the GFP fusion in this configuration does not alter the functionality of the channel expressed in HEK-293 cells whereas a fusion protein that is very close to the functional domain (e.g., channel pore, catalytic center, or ligand-binding site) is more likely to disrupt the structure or biological activity of the protein (Hoffmann et al., 2010). In some cases, the maturation time of fluorescent proteins may be a limiting factor especially when studying rapid protein-ligand or protein-protein interactions (Snapp, 2005). While the use of a smaller label such as the membrane-permeant fluorogenic biarsenical dye fluorescein arsenical hairpin binder (F1AsH) or the alternative red-shifted resorufin arsenical hairpin binder analog (ReAsH) can conceivably overcome structural and the concomitant functionality concerns, these epitopes require labeling with biarsenical dyes that may be toxic to the cells. In addition, non-specific binding of the dye to the hydrophobic regions of the membrane in the cells is commonly associated to the problem of high background staining which may be overcome by an additional washing procedure upon labeling (Martin et al., 2005). Still, in our case, we did not notice a significant improvement in the staining efficiency even after washing. Furthermore, the washing procedure involving thiol-containing reagents may damage the cells and alternative protein functions (Langhorst et al., 2006). The contrast and cytotoxicity issues commonly associated with the Lumio™ reagents have been discussed elsewhere (Hoffmann et al., 2010).

### Assessment of commonly used transfection reagents

**Figure 3:**
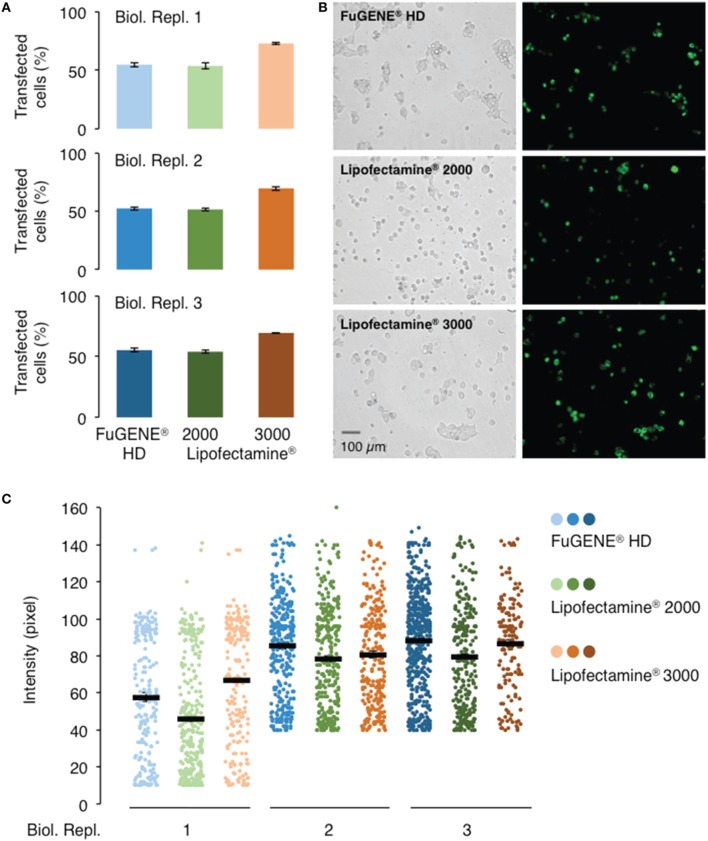
**Comparison of different transfection reagents on the transfection and expression efficiencies of AtGORK-EmGFP in HEK-293 cells**. HEK-293 cells transfected with *AtGORK-EmGFP* were used to examine the performance of different transfection reagents in a “reverse” format (see Section Procedure for definition). In general, Lipofectamine^®^ 3000 outperforms Lipofectamine^®^ 2000 and FuGENE^®^ HD in both transfection efficiency **(A,B)** and protein expression **(C)**. Lipofectamine^®^ 2000 and FuGENE^®^ HD have comparable transfection efficiencies but lower than that of Lipofectamine^®^ 3000. FuGENE^®^ HD seems to yield protein expression levels comparable to that of Lipofectamine 3000. A typical 20 × HEK cell image contains 2560 × 1920 pixels and is converted to an 8-bit byte image that on a scale of 0–255 using ImageJ. The lower limit cut-off that represents appreciable and above background fluorescence, was set at 10 pixels for biological replicate 1 but 40 pixels for replicates 2 and 3. Transfection efficiency and protein expression of more than 200 cells in each viewing area were analyzed (see Section Procedure for analysis).

In order to evaluate the performance of current commercially available transfection reagents, we transiently transfected the 293FT cells with the *AtGORK-EmGFP* construct using the standard lipid-mediated transfection reagents, Lipofectamine^®^ 2000, Lipofectamine^®^ 3000, and non-liposomal FuGENE^®^ HD formulation in a “reverse” format (see Section Procedure for definition). Lipofectamine^®^ 3000 consistently outperform Lipofectamine^®^ 2000 and FuGENE^®^ HD in transfection efficiency, achieving approximately a 70% transfection rate with AtGORK-EmGFP in comparison to the other two reagents which achieved a rate of about 50% (Figures 3A,B). The transfected cells exhibit an intensity of green fluorescence that corresponds to the expression levels of AtGORK-EmGFP and is consistently higher when transfected using the Lipofectamine^®^ 3000 or FuGENE^®^ HD as compared to the Lipofectamine^®^ 2000 (Figure 3C). This suggests that while FuGENE^®^ HD yields lower transfection efficiency than Lipofectamine^®^ 3000, the protein expression level is comparable to that of Lipofectamine^®^ 3000.

We observed a marked decline in transfection efficiency when the reagents are left at room temperature for prolonged periods of time. We note that the Lipofectamine^®^ and FuGENE^®^ HD formulations should be stored at 4°C and should be used immediately after the reagents have been equilibrated to room temperature. Furthermore, it is important to incubate these transfection reagents for the durations and at conditions recommended by their respective manufacturers prior to addition to cells (Table 1). We also noted that these incubation times and conditions should be adhered to as strictly as possible since prolonged incubation times decrease transfection efficiencies. Our results suggest that Lipofectamine^®^ 3000 outperforms Lipofectamine^®^ 2000 and FuGENE^®^ HD in transfection efficiency and/or protein expression, which is consistent with the claims of the manufacturer who reported it for HEK-293, HeLa, LNCaP, HepG2, and A549 cell lines, the superiority of Lipofectamine^®^ 3000.

### Comparison of different transfection protocols

**Figure 4:**
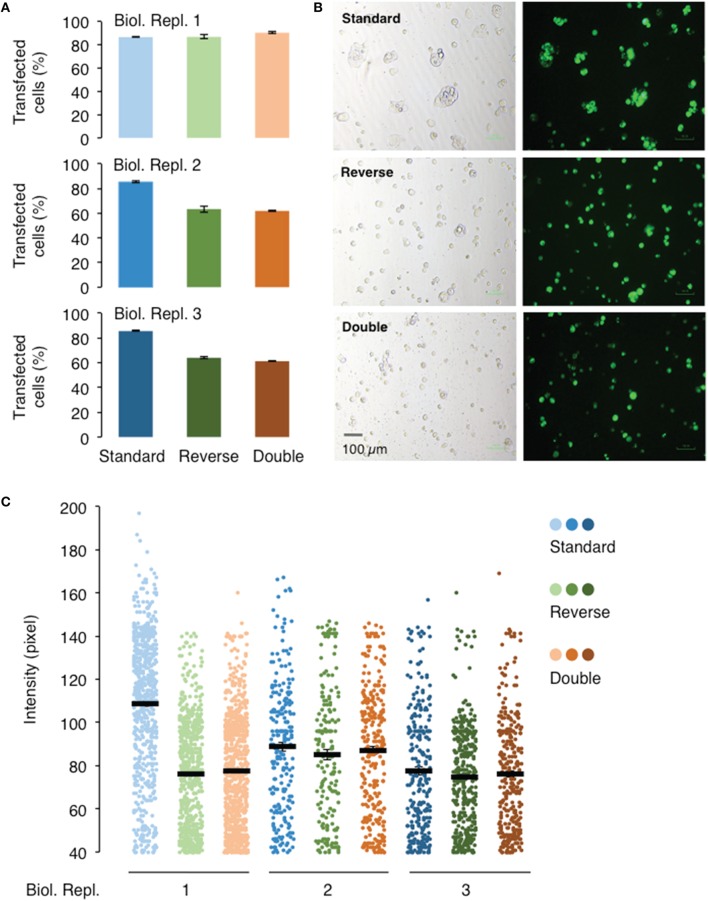
**Effect of different transfection methods on the transfection and expression efficiencies of AtGORK-EmGFP in HEK-293 cells**. HEK-293 cells transfected with *AtGORK-EmGFP* were used to examine the performance of different transfection format: “standard,” “reverse,” and “double” (see Section Procedure for definition) using the Lipofectamine^®^ 3000 reagent. The “standard” transfection method outperforms both the “reverse” and “double” transfection format respectively, in 2 out of 3 biological repeats in terms of transfection efficiency **(A,B)**. The “standard” transfection protocol yields higher protein expression levels in only 1 biological replicate but yield comparable expression levels to the other transfection methods in replicates 2 and 3 **(C)**. A typical 20 × HEK cell image contains 2560 × 1920 pixels and is converted to an 8-bit byte image that is on a scale of 0–255 using ImageJ. The lower limit cut-off representing appreciable and above background fluorescence, was set at 10 pixels for 40 pixels. Transfection efficiency and protein expression of more than 100 cells in each viewing field were analyzed (see Section Procedure for analysis).

The three transfection protocols, “standard,” “reverse,” and “double” (see Section Procedure for definition), were also evaluated for their transfection and expression efficiencies of AtGORK channel in 239FT cells using the Vivid Colors™ pcDNA™6.2/EmGFP-DEST Gateway^®^ plasmid and Lipofectamine^®^ 3000 reagent. The “reverse” and “double” transfections have similar transfection efficiencies and protein expressions levels (Figure 4). Their transfection efficiency of around 60% is lower than that achieved with the “standard” transfection (80%) in two of the three biological replicates (Figures 4A,B) while only in one of three biological replicates, the “standard” transfection had higher protein expression as deduced from the fluorescence intensities (Figure 4C).

The “standard” transfection protocol while resulting in better transfection efficiency, requires one additional day as compared to the “reverse” transfection because the cells are seeded overnight before the addition of Lipofectamine^®^ 3000 reagent and the *AtGORK-EmGFP* plasmid. Since the cells are allowed to grow for an additional day in the “standard” transfection method, more cells are clumped together and this may complicate single-cell selection for patch-clamp studies. These factors, such as the transfection efficiency, time and cell morphology should be considered case-by-case basis depending on the intended application. As for the transfection of membrane proteins or ion channels in HEK-293 cells, we recommend the “reverse” transfection protocol starting with a seed number of approximately 250,000 cells in a total volume of 2 mL. The “double” transfection protocol may be useful for the transfection of large plasmids (>10 kb) or choice of cell lines that usually have low transfection efficiency in single transfection. However, we noted that in the transfection of 293FT cells with *AtGORK-EmGFP*, the efficiency did not improved with the double transfection protocol since an optimal uptake of plasmids may already have been achieved in single transfection.

### A general protocol for the expression of membrane proteins in HEK-293 cells for current-voltage measurements

We detail optimized experimental parameters (Table 2) for high expression of AtGORK channel in 293FT cells for single-cell applications such as electrophysiological characterization and describe the experimental workflow (Figure S4). This authoritative step-by-step protocol serves as a good platform from that, users can conduct informed and less risky targeted optimizations, thus benefiting first time users or those with proteins of unknown properties. Based on this protocol, rational changes can be made in particular to the design of expression vectors with suitable identification tags, as well as the choice of transfection reagents and experimental parameters in order to cater for the expression of membrane proteins in general and/or for other functional characterizations.

In summary, we have described an optimized protocol that serves as a general guide for the transient transfection and expression of a membrane protein in HEK-293 cells for functional characterizations in general and single-cell applications in particular. We demonstrate that the use of a GFP-fused expression construct, Vivid Colors™ pcDNA™6.2/EmGFP-DEST Gateway^®^ vector at 2.5 μg using Lipofectamine^®^ 3000 reagent in a “reverse” format yields a high transfection efficiency, protein expression level as well as optimal detection of AtGORK channels in 293FT cells. We also recommend experimental parameters amendable for other applications including cautionary steps associated with this protocol. We believe that this optimized method will facilitate the physiological and cellular characterization of complex membrane proteins.

## Author contributions

Both AO and AW conceived and designed this project. AO, LE, and FL-C conducted the experiments. All authors contributed to the data analyses and writing of the manuscript.

### Conflict of interest statement

The authors declare that the research was conducted in the absence of any commercial or financial relationships that could be construed as a potential conflict of interest.

## Supplementary material

Figure S1 **Design of AtGORK (At5G37500) plasmid construct. (A)** Domain organization of full-length *Arabidopsis thaliana* GORK (AtGORK) (At5G37500) channel and the insertion of the AtGORK gene into pcDNATM6.2/cLumioTM-DEST. **(B)** Domain organization of full-length *Arabidopsis thaliana* GORK (AtGORK) (At5G37500) channel and the insertion of the AtGORK gene into Vivid Colors™ pcDNATM6.2/EmGFP-DEST Gateway^®^ vector.

Figure S2 **Comparison of different amount of AtGORK-cLumio™ plasmid for transfection**.

Figure S3 **mRNA transcript of AtGORK-cLumio™ heterologously expressed in HEK-293 cells**.

Figure S4 **Experimental workflow for heterologous expression of membrane proteins in HEK-293 cells for electrophysiological characterizations. (A)** Plasmid design and label selection. **(B)** Transfection and expression. **(C)** Fluorescent imaging and quantification. **(D)** Current-voltage measurement and data analysis.
