# Supplementary figures and images for: A Guide to Transient Expression of Membrane Proteins in HEK-293 Cells for Functional Characterization
Source: Front Physiol. 2016 Jul 19;7:300. doi: 10.3389/fphys.2016.00300 (PMC4949579; doi:10.3389/fphys.2016.00300)

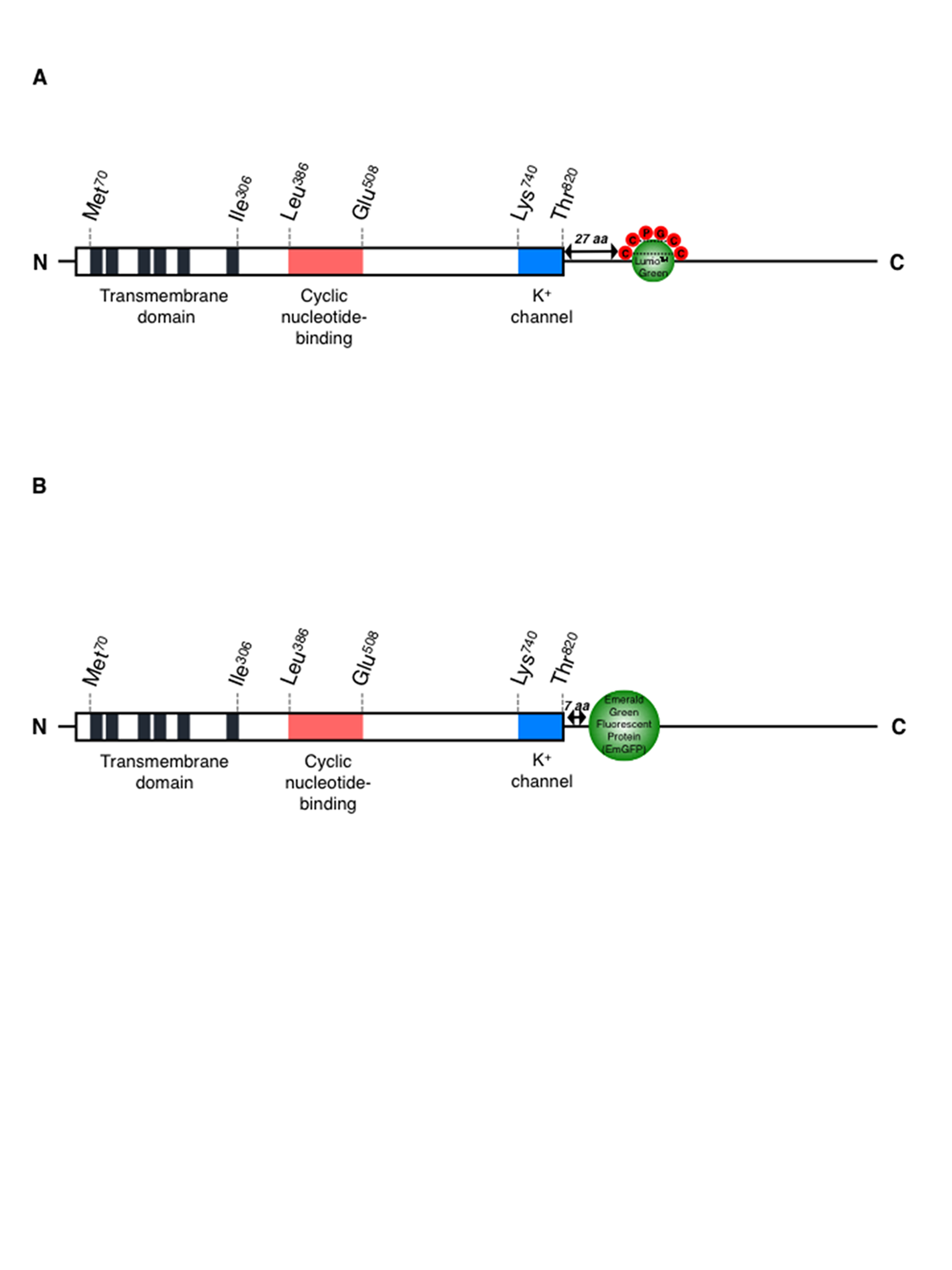

Supplement: Figure S1 — Design of AtGORK (At5G37500) plasmid construct. (A) Domain organization of full-length Arabidopsis thaliana GORK (AtGORK) (At5G37500) channel and the insertion of the AtGORK gene into pcDNATM6.2/cLumioTM-DEST. (B) Domain organization of full-length Arabidopsis thaliana GORK (AtGORK) (At5G37500) channel and the insertion of the AtGORK gene into Vivid Colors™ pcDNATM6.2/EmGFP-DEST Gateway® vector. [file Image1.TIFF]

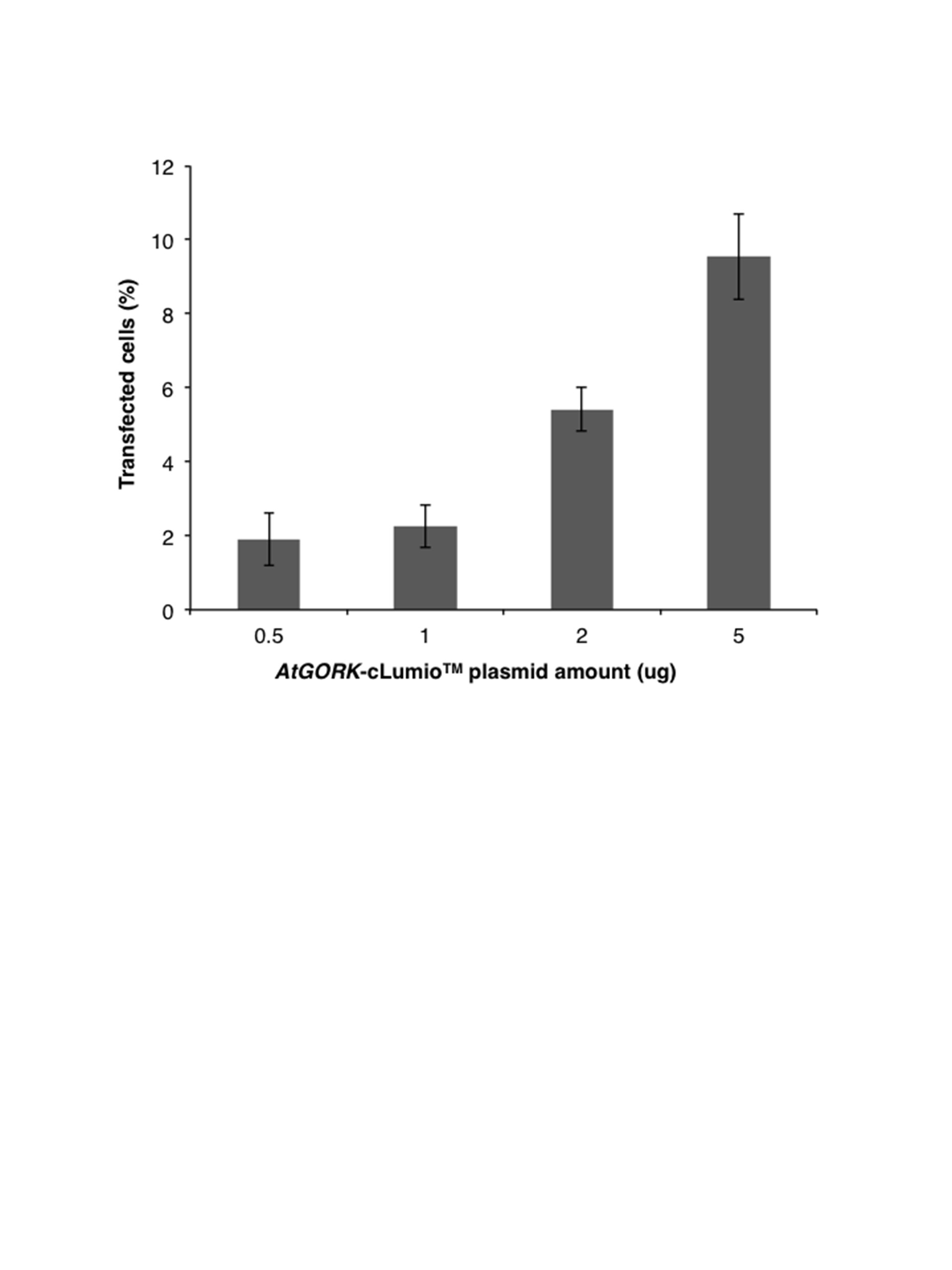

Supplement: Figure S2 — Comparison of different amount of AtGORK-cLumio™ plasmid for transfection. [file Image2.TIFF]

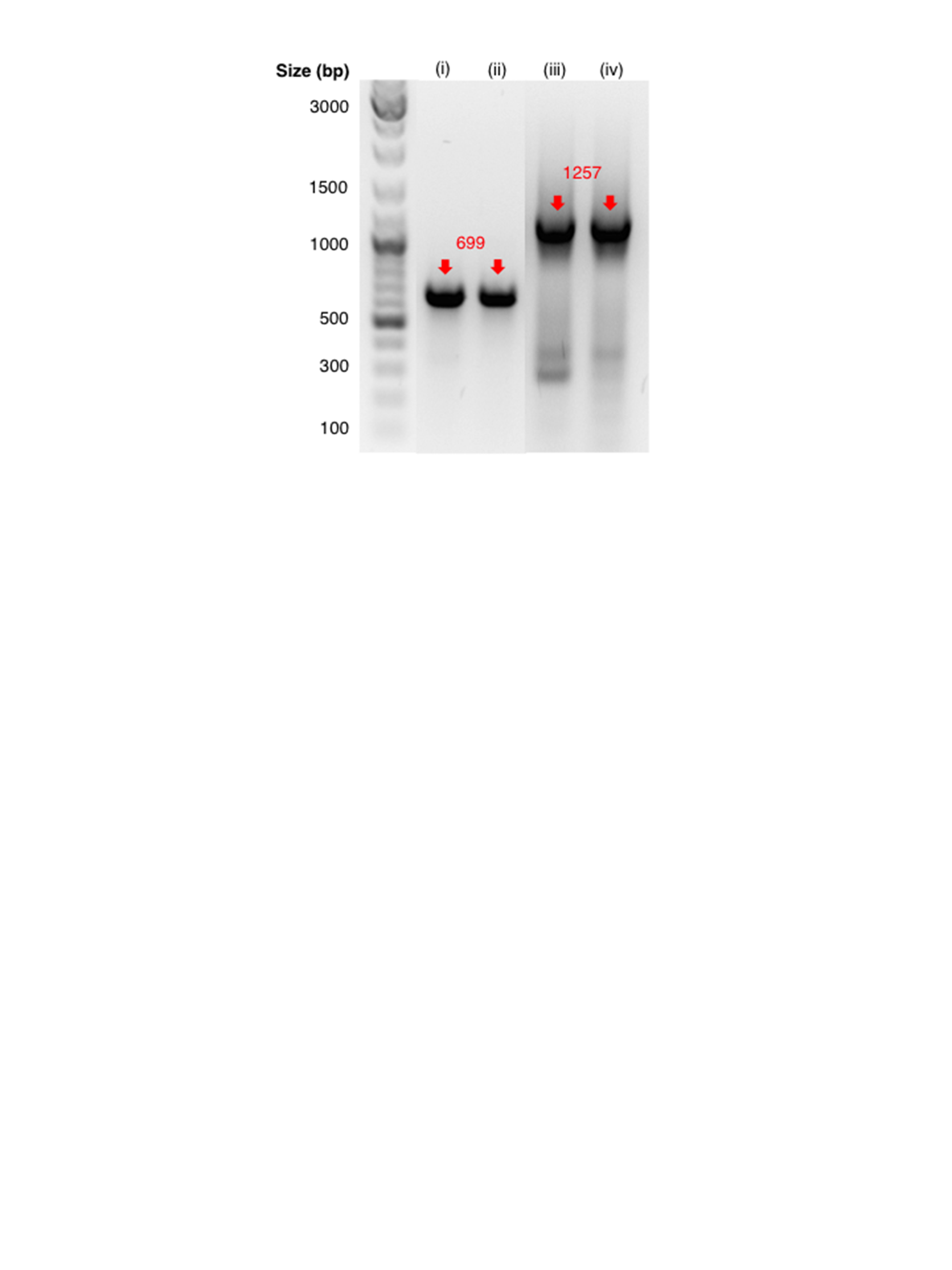

Supplement: Figure S3 — mRNA transcript of AtGORK-cLumio™ heterologously expressed in HEK-293 cells. [file Image3.TIFF]

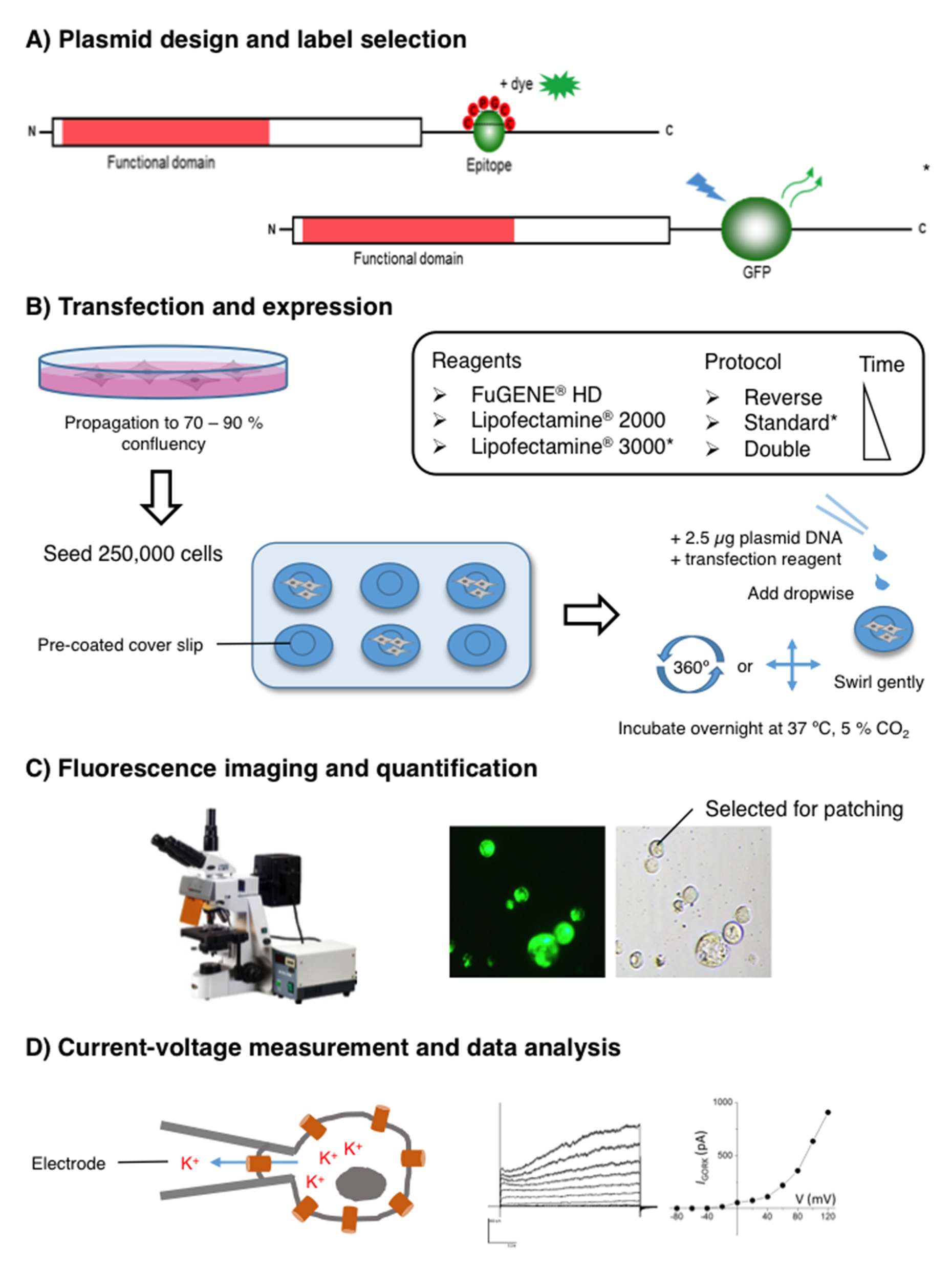

Supplement: Figure S4 — Experimental workflow for heterologous expression of membrane proteins in HEK-293 cells for electrophysiological characterizations. (A) Plasmid design and label selection. (B) Transfection and expression. (C) Fluorescent imaging and quantification. (D) Current-voltage measurement and data analysis. [file Image4.TIFF]
